# Supplementary material for: Comprehensive Annotation and Functional Exploration of MicroRNAs in Lettuce
Source: Front Plant Sci. 2021 Dec 24;12:781836. doi: 10.3389/fpls.2021.781836 (PMC8739914; doi:10.3389/fpls.2021.781836)
Supplement: Supplementary file 2 [file Data_Sheet_8.ZIP › GSM2230748.plot/Lsa-miR166g_Lsat_1_v5_gn_3_10861.1_514_TPlot.pdf]

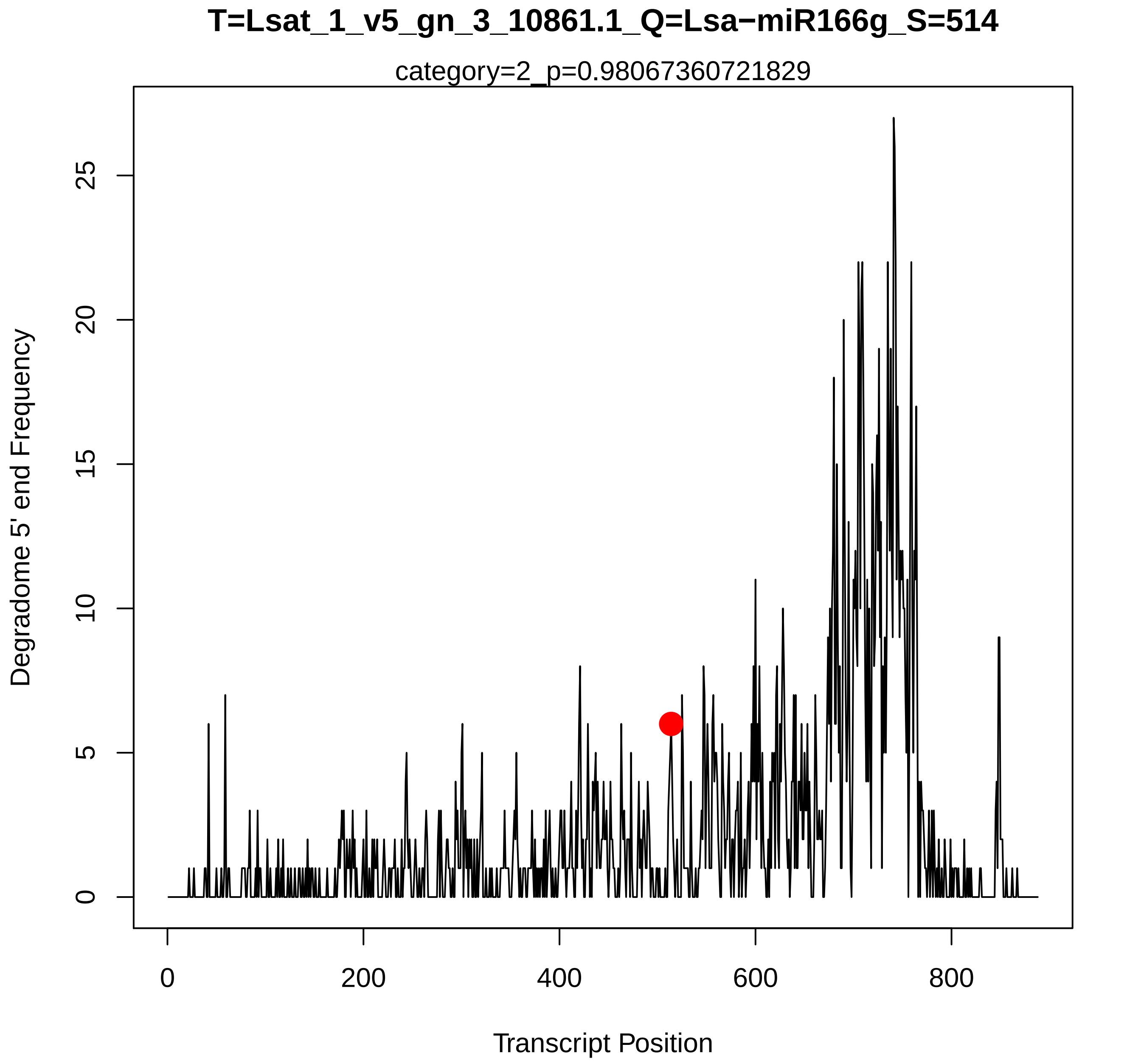

T=Lsat_1_v5_gn_3_10861.1_Q=Lsa−miR166g_S=514
category=2_p=0.98067360721829
Degradome 5' end Frequency
0
5
10
15
20
25
0
200
400
600
800
Transcript Position
